# Psychometric properties of the Functional Assessment of Cancer Therapy–Lung (FACT-L) scale to measure health-related quality of life

**DOI:** 10.1186/s41687-026-01164-4

**Published:** 2026-07-31

**Authors:** Jose A. Carreno, Luis Fontibón, Carlos Carvajal, Jorge Rugeles, Alicia Quiroga, Ricardo Buitrago, Jose Venegas, Ricardo Sanchez

**Affiliations:** 1 Instituto Nacional de Cancerología, Bogotá, Colombia; 2 National University of Colombia, Bogotá, Colombia; 3 Clínica IMAT Oncomédica Auna, Montería, Colombia; 4 Hospital Pablo Tobon Uribe, Medellín, Colombia; 5 Clinica de Marly, Bogotá, Colombia; 6 Hospital Alma Máter de Antioquia, Medellín, Colombia

**Keywords:** Health-Related Quality of Life, Lung neoplasms validation studies, Oncology patients, Lung cancer

## Abstract

**Purpose:**

To validate the Functional Assessment of Cancer Therapy–Lung (FACT-L) scale and assess its psychometric properties in patients with lung cancer in Colombia.

**Methods:**

The analysis included exploratory and confirmatory factor analysis, internal consistency (248 patients diagnosed with lung cancer), test–retest reliability (56 patients evaluated at two different time points), discriminant, convergent and divergent validity.

**Results:**

Factor analysis revealed a two-dimensional structure within the lung cancer-specific subscale (LCS). Cronbach’s alpha was 0.91, ω McDonald 0.92 and Guttman’s λ2 0.92 for the FACT-L. Lin’s concordance correlation coefficient indicated test–retest reliability (0.8, 95% CI: 0.69–0.88) adequate for uses of the tool. Convergent validity assessed through Spearman’s rho correlation coefficient was 0.64 (95% CI: 0.56–0.71, *p* < 0.001). In divergent validity, the result was a *rho* − 0.432 (P = < 0.001); in discriminant validity was a *rho* of 0.141 (*P* = 0.027).

**Conclusion:**

Internal consistency analyses demonstrated strong inter-item correlations and high homogeneity. Discriminant, convergent, and divergent validity support the scale’s ability to distinguish between conceptually distinct constructs, and test–retest reliability confirmed temporal stability. The FACT-L scale is a valid and reliable instrument for assessing health-related quality of life in patients with lung cancer in the Colombian context.

**Supplementary Information:**

The online version contains supplementary material available at https://doi.org/10.1186/s41687-026-01164-4.

## Introduction

As of 2022, lung cancer remains the leading cause of cancer-related mortality worldwide, accounting for approximately 18.7% of all cancer deaths, with over 1.8 million fatalities annually. It has also emerged as the most frequently diagnosed cancer globally, representing 12.4% of all new cancer cases and nearly 2.5 million new diagnoses. Currently, one in every eight new cancer cases are attributed to lung cancer [1].

The clinical presentation of lung cancer is often insidious. Common symptoms include cough, hemoptysis, dyspnea, chronic airway inflammation, chest pain, asthenia, adynamia, and anorexia. However, these manifestations are typically non-specific, and a significant proportion of patients remain asymptomatic during the early stages of the disease. This lack of early clinical indicators poses a substantial barrier to timely diagnosis and intervention [2–6].

In Colombia, this diagnostic delay is reflected in the advanced stage at which most lung cancer cases are identified. Recent data indicate that between 10.2% and 15.6% of cases are diagnosed at stage III, while 67.7% to 79.3% are detected at stage IV [7, 8]. This late-stage presentation significantly limits curative treatment options and contributes to the high mortality associated with the disease [9–11]. More than half of patients succumb to the illness within an average of 9.2 months following diagnosis. Despite advances in therapy, the five-year survival rate for lung cancer has shown minimal improvement and remains below 6% [12–17].

Patients with lung cancer have been consistently reported to experience lower health-related quality of life (HRQoL) compared to individuals with other malignancies. Those presenting with more severe symptoms tend to report even poorer HRQoL Outcomes [18]. The burden of symptoms has been shown to significantly impact emotional well-being, further compromising overall quality of life in this population [19, 20].

In the context of advanced-stage lung cancer, the primary goals of treatment are to prolong survival and enhance quality of life through effective symptom management.

There is growing evidence supporting the routine integration of HRQoL assessments into cancer care. Regular monitoring of HRQoL during treatment has been shown to facilitate better management of treatment-related toxicity and enable earlier detection of disease progression [21].

To assess HRQoL in cancer patients, a variety of instruments have been developed to provide objective, valid, and reliable quantification of this multidimensional construct. For lung cancer specifically, there are several instruments, including the FACT-L, the EORTC QLQ-LC29, and the Lung Cancer Symptom Scale (LCSS). The EORTC QLQ-LC29 is frequently used in clinical trials due to its comprehensive coverage of physical and functional symptoms such as fatigue, pain, and nausea. In contrast, the FACT-L adopts a more patient-centered approach, emphasizing emotional and social dimensions of well-being [22].

Evidence suggests that FACT-L demonstrates a stronger internal consistency and test-retest reliability, along with a broader range of psychometric strengths. The FACT-L has fewer items making it more feasible for use in time-constrained clinical settings, potentially improving patient compliance. Importantly, the FACT-L has also been recognized as a valuable prognostic tool, with demonstrated utility in predicting survival outcomes in lung cancer patients [23–25]. Given these advantages, this study sought to validate the FACT-L version 4 scale and assess its psychometric properties in a cohort of lung cancer patients in Colombia.

## Patients and methods

This study was conducted between 2021 and 2024 across five leading cancer care institutions in Colombia: The National Cancer Institute and Clínica Marly in Bogotá, Hospital Pablo Tobón Uribe and Hospital Alma Mater in Medellin, and Clínica IMAT Oncomédica in Monteria. These institutions, both public and private, serve as national reference centers for oncology and are in regions where the same dialect is used without variants of Spanish language with a high prevalence of lung cancer [26]. To identify eligible participants, clinical records from specialized outpatient services in pulmonology, thoracic surgery, and oncology were reviewed. Inclusion criteria comprised adults (≥ 18 years) with a histologically confirmed diagnosis of primary lung cancer at any stage, Colombian nationality, absence of cognitive impairment, and voluntary consent to participate.

The first measure of the Functional Assessment of Cancer Therapy – Lung (FACT-L) scale was administered in person or via telephone interviews according to the availability or convenience of each patient. The next measurements, which were every three months until 24 months or death if it occurred first, were via telephone. Clinical data were extracted directly from patient records at each participating institution. Eligibility verification was performed by the principal investigator designated in each institution.

The data were registered in the Redcap platform, with dual-entry verification by two team members to ensure data integrity. An independent monitoring team oversaw the transcription process and quality control of the dataset.

## The Functional Assessment of Cancer Therapy – Lung (FACT-L)

The FACT-L scale was originally developed by the FACIT.org group and first validated in 1995, and it’s available in 53 languages. including Spanish, therefore cross cultural validation is not required. Today FACT-L has undergone validation in at least five different international contexts [27]. The FACT-L Version 4 consists of 36 items, integrating the four core domains of the FACT-G (Physical Well-Being [PWB], Social/Family Well-Being [SWB], Emotional Well-Being [EWB], and Functional Well-Being [FWB]) along with a 10-item Lung Cancer Subscale (LCS). Items are rated on a 5-point Likert scale (0 = Not at all to 4 = Very much), except for one item (Q3: “Have you been a smoker?”). Total scores range from 0 to 136, with higher scores indicating better HRQoL. Additionally, the Trial Outcome Index (TOI), derived from the sum of PWB, FWB, and LCS scores, is often used in clinical trials to assess the physical aspects of HRQoL [28], with a score range of 0 to 84 LCS score overtime was examined in relation to changes in ECOG performance and Charlson Comorbidity Index (CCI) scores [29].

The Korean version demonstrated strong convergent and divergent validity when compared with the Functional Living Index-Cancer (FLIC) and abbreviated versions of the Profile of Mood States (POMS) [30]. In China, factor and correlation analyses supported the construct and criterion-related validity of the scale, particularly in comparison with the Quality-of-Life Instruments for Cancer Patients–Lung Cancer (QLICP-LU). Discriminant validity was also confirmed through age group comparisons [31]. In Japan, internal consistency was assessed using Cronbach’s alpha and factor analysis, while clinical validity was evaluated by comparing patient groups with varying symptom burdens. Although sensitivity to change was explored, small sample sizes limited statistical significance over time [32]. The Brazilian version included both the FACT-L and the FACT-Lung Symptom Index (FLSI), confirming their reliability and ease of use in clinical settings [33]. The Turkish version reported higher Lung Cancer Subscale (LCS) scores than the original validation, with internal consistency (Cronbach’s α) ranging from 0.60 to 0.84 [34]. Across these versions, the intraclass correlation coefficient (ICC) for test-retest reliability ranged from 0.52 to 0.96, with the Brazilian version showing the highest reliability (ICC: 0.78–0.96), followed by the Chinese (0.76–0.82) and Korean (0.52–0.84) versions. Test-retest reliability was not assessed in the Turkish version, while the Japanese version reported item-level correlation coefficients above 0.50. Internal consistency (Cronbach’s α) ranged from 0.56 to 0.84 across studies [32].

For the present validation study, formal authorization was obtained from the FACIT.org group to use the Spanish version of the FACT-L scale.

### Statistical analysis

For the validation of the FACT-L, the classical test theory (CTT) framework was applied. This included construct validity via exploratory (EFA) and confirmatory factor analysis (CFA), internal consistency, discriminant, convergent and divergent validity.

Construct validity was assessed through exploratory factor analysis (EFA) and confirmatory factor analysis (CFA), which were conducted using data from 248 patients. The EFA was performed using ordinal factor analysis techniques to explore the internal structure of the LCS and to identify underlying dimensions. The suitability of the data for factor analysis was confirmed using the Kaiser-Meyer-Olkin (KMO) measure of sampling adequacy and Bartlett’s test of sphericity [35]. The number of factors to retain was determined using multiple criteria: eigenvalues greater than 1, parallel analysis, and scree plot inspection (see supplemental Fig. 1S) [36]. A polychoric correlation matrix was used to account for the ordinal nature of the Likert-type items [37]. In accordance with the administration and scoring guidelines provided by FACIT.org, two items: B5 (“I am bothered by hair loss”), Q3 (“Have you been a smoker?“) and L5 (“I regret smoking”) were excluded from the analysis, leaving seven items from the Lung Cancer Subscale (LCS) for evaluation. Consistent with the findings of Cella et al. [38], the exclusion of these items improved internal consistency (Cronbach’s α increased from 0.53 to 0.68). Factor extraction was followed by both orthogonal and oblique rotation methods to identify the most clinically interpretable factor solution [39]. To empirically confirm the theoretical structure of the construct and the grouping of latent variables, a CFA was conducted using structural equation modeling.

The CFA was applied to the sample of 248 patients employed the diagonally weighted least squares (DWLS) estimation method, which is appropriate for ordinal data and non-normal distributions [40]. Model fit was evaluated using multiple indices: the chi-square to degrees of freedom ratio (χ²/df), root mean square error of approximation (RMSEA), Bentler-Bonett non-normed fit index (NNFI), comparative fit index (CFI), goodness-of-fit index (GFI), and standardized root mean square residual (SRMR). Acceptable model fit was defined as χ²/df < 3, RMSEA < 0.08, SRMR < 0.08, and NNFI, CFI, IFI, TLI, and McDonald’s Fit Index (MFI) values > 0.90 [41]. All statistical analyses were performed using psych packages in R [42].

The consistency between repeated measurements with the same instrument (test-retest reliability) was evaluated in a subsample of 56 patients who completed the FACT-L scale at two different time points which, according to the recommended standard in classical psychometric research, is less than 2 weeks. Agreement between the two observations was evaluated using Lin’s concordance correlation coefficient (ρ c), with values above 0.80 considered acceptable [43] using a significance level of 5% for testing hypotheses, additionally, a Bland–Altman plot was generated to visually assess the level of agreement between those two repeated measurements.

Internal consistency was assessed using Cronbach’s alpha (α), McDonald’s omega (ω), and Guttman’s lambda-2 (λ²) coefficients. Values exceeding 0.70 were considered indicative of acceptable reliability [44]. This analysis was conducted with 248 patients; the sample size achieves a power greater than 88% to detect a difference of 0.15 between a null hypothesis correlation of 0.65 and the alternative hypothesis correlation of 0.8 using a two-sided hypothesis test with a significance level of 0.05. Further analysis included item-withdrawal procedures for both Cronbach’s α and McDonald’s ω to evaluate the impact of individual items on overall scale consistency [45].

Convergent validity was assessed by examining the correlation between the scores of the FACT-G scale and the Lung Cancer Subscale (LCS) using Pearson or Spearman’s rho correlation coefficient [46]. This analysis was conducted with 248 patients; the sample size achieves a power greater than 90% to detect a difference of 0.1 between an alternative hypothesis correlation of > 0.4 and the null hypothesis correlation of < 0.30; correlation values greater than 0.40 were considered acceptable. These analyses provided evidence of internal consistency and alignment between related constructs within the FACT-L instrument.

Divergent validity was assessed to determine negative correlations between the Lung Cancer Subscale (LCS) and scales that measure different constructs, such as Eastern Cooperative Oncology Group (ECOG) performance status. ECOG is a clinical tool that assesses a patient’s functional status, especially in oncology settings. It describes a patient’s level of functioning in terms of their ability to care for themselves, daily activities, and physical ability (walking, working, etc.), ranging from being fully active (ECOG 0) to death (ECOG 5) at ecog-acrin.org/scale. A negative correlation is expected between LCS and ECOG. Pearson correlation coefficient or Spearman’s rho < 0.30 is considered indicative of acceptable divergent validity [47].

Discriminant validity was assessed to determine a correlation between LCS and the Charlson Comorbidity Index (CCI). The CCI is primarily used to predict mortality based on the presence and severity of comorbidities. Each comorbidity has specific weights. The sum of these weights generates a comorbidity score, which means higher scores are related to a higher risk of mortality to predict 10-year survival [48]. A low or zero correlation is expected between LCS and CCI, since they measure different concepts: perceived well-being vs. comorbidity burden, quality of life levels; generally, it is not expected to be influenced by disease burden [46]. This analysis was conducted with 248 patients; the sample size achieves a power greater than 88% to detect a difference of 0.15 between an alternative hypothesis correlation of < 0.3 and the null hypothesis correlation of 0.45, considering Pearson or Spearman’s rho correlation coefficients. All statistical analyses were performed using R.

This study was registered in the Protocol Registration and Results System (PRS) under the identifier NCT06900283 21/March/ 2025. Ethical approval was obtained from the institutional review boards of all participating centers. Informed consent was obtained from all participants prior to enrollment, in accordance with the ethical principles outlined in the Declaration of Helsinki.

## Results

This validation study of the FACT-L scale was conducted across five cancer care institutions in Colombia. Most participants (77.8%) were recruited from hospitals located in the Andean region (Bogotá and Medellín), with the remaining participants from the Caribbean region (Montería).

A total of 248 participants who met the inclusion criteria were enrolled in the study. The median age was 65,8 years (IQR = 15,6), with a predominance of female participants *n* = 152 (62.2%). Regarding the educational attainment, *n* = 45 (18.1%) reported having completed higher education, most of the sample belonged to a low socioeconomic (status 1and 2) *n* = 144 (58,1%. According to the marital status, just over half reported being in a stable partnership (married and common-law marriage), *n* = 130 (52%). Regarding occupation, *n* = 94 (38%) were unemployed at the time of the study.

Non-small cell lung cancer (NSCLC) was the predominant histological type, diagnosed in 238 patients (96.3%). Among these, adenocarcinoma was the most frequent subtype, accounting for 157 cases (86.7%). Most patients, *n* = 194 (78.1%), were diagnosed at advanced stages (III–IV), underscoring the persistent challenges in early detection and the absence of a national lung cancer screening program. Exposure to cigarette smoke was reported by 107 participants, of whom 77 were former smokers with cessation periods exceeding 30 years; despite existing tobacco control policies and public health campaigns. Notably, 83 of these individuals were former smokers with a smoking history exceeding 30 years. The main comorbidities were metastatic disease *n* = 211 (85,1%), chronic respiratory disease *n* = 45 (18,1%), diabetes *n* = 43 (17,3%), and heart disease *n* = 15 (6,0%). The mean Charlson Comorbidity Index (CCI) was 6.4 (SD 2,84), and *n* = 200 patients (80.6%) of participants had an Eastern Cooperative Oncology Group (ECOG) performance status of 0–1, indicating relatively preserved functional status. Detailed sociodemographic and clinical characteristics are presented in Tables 1a and 1b.

**Table 1a:** Demographics

	Total	%
**Age (median, IQR)**	65,8 (15,6)	
**Women (%)**	152	62,2%
**Men (%)**	96	38,7%
**Educational attainment**		
Complete primary school	48	19%
Incomplete bachelorship	46	18%
Higher education	45	18%
Incomplete primary school	43	17%
Bachelorship	34	14%
Graduate	23	9%
No education	9	4%
**Marital status**		
Married	90	36%
Bachelor	54	22%
Widower	45	18%
Common-law marriage	40	16%
Separate	19	8%
**Occupation**		
Unemployed	94	38%
Retiree	47	19%
household activities	45	18%
Independent worker	35	14%
Employee	27	10%
Student	1	0%
Other	1	0%
Socioeconomic Status *		
1	49	20%
2	95	38%
3	70	28%
4	23	9%
5	13	4%

*The levels indicate the inverse level of poverty (e.g. 1 very poor, 5 better income)

**Table 1b:** Clinical features

Histological classification	*n*				%	
Non-small cell lung cancer	238				96,3%	
Adenocarcinoma	157				86,7%	
Squamous cell carcinoma	23				12,7%	
Other	58				24,4%	
Small cell lung cancer	10				4,0%	
**Exposure to carcinogens**						
Cigarette smoke	107				43,1%	
Biomass	69				27,8%	
Asbestos	6				2,4%	
Arsenic	6				2,4%	
Paints	5				2,0%	
None	41				16,5%	
No data	14				5,6%	
**Stage**						
I	23				9,3%	
II	16				6,5%	
III	41				16,5%	
IV	153				61,7%	
No data	15				6%	
**ECOG Perfomance Status**						
Grade 0	126				50,8%	
Grade 1	74				29,8%	
Grade 2	39				15,7%	
Grade 3	6				2,4%	
Grade 4	3				1,2%	
**Karnofsky Scale**						
40	2				0,8%	
50	7				2,8%	
60	12				4,8%	
70	20				8,1%	
80	28				11,3%	
90	67				27,0%	
100	77				31,0%	
No data	35				14,1%	
**Charlson Comorbidity Index (CCI)**						
Absence or low comorbidity (0–1)	1				0,4%	
Moderate comorbidity (2)	48				19,4%	
High comorbidity (> 3)	199				80,2%	
Mean	6,64				2,84 (SD)	
**Main Comorbidities** ¥						
Metastatic disease						
Yes	211				85,1%	
No	37					
Chronic respiratory disease						
Yes	45				18,1%	
No	203					
Diabetes						
Yes	43				17,3%	
No	205					
Heart disease						
Yes	15				6,0%	
No	233					
Cerebrovascular disease						
Yes	7				2,8%	
No	241					
Treatments *						
Surgery	123				49,2%	
Chemotherapy	107				42,8%	
Palliative care	80				32,0%	
Immunotherapy	66				26,4%	
Radiotherapy	63				25,2%	
Targeted therapy	51				20,4%	
Concomitant chemotherapy radiotherapy	8				3,2%*	

¥ Refers to the comorbidities (in some cases, more than one) after the first survey

*Refers to the treatment (more than one) received during or near the completion of the survey

### Scale scores

The mean scores for each domain of the FACT-L scale were as follows: Physical Well-Being (PWB) 18.5 (SD = 6.5), Social/Family Well-Being (SWB) 20.1 (SD = 4.2), Emotional Well-Being (EWB) 17.7 (SD = 4.6), Functional Well-Being (FWB) 16.2 (SD = 5.3), and Lung Cancer Subscale (LCS) 19.2 (SD = 5.0). The overall mean FACT-L score was 91.9 (SD = 19.6), within a range of 0 to 136. The mean scores for LCS, FACT-TOI, and FACT-L showed differences when they were stratified by ECOG, CCI, and stage. For instance, in LCS, the mean for ECOG 0 and 4 was 21.3 (SD 4.37) and 13.0 (SD 7.93), respectively. Similarly, in FACT-L, the mean for Stage I and IV was 107.4 (SD 16.2) and 88.1 (SD 19.0) respectively. The Quality of Life decreased when the functionality was low, high comorbidity, and advanced stages (see supplemental Table 3).

### Exploratory factor analysis

Assumptions for factor analysis were verified prior to model estimation. The Kaiser-Meyer-Olkin (KMO) measure of sampling adequacy was 0.72. Bartlett’s test of sphericity was statistically significant (χ² = 332.8, *p* < 0.001), confirming the Matrix factorability. Given the ordinal nature of the data, a polychoric correlation matrix (PCM) was constructed to model the relationships between the items. To determine the optimal number of factors to extract, multiple criteria were analyzed: acceleration factor, Scree (SE), Scree (R2), Very Simple Structure (VSS), complexity 1, Velicer, Minimum Average Partial correlation (MAP). Solutions with one or two factors allowed for factor structures with more than two items, and both orthogonal and oblique rotations were used; the quartimax rotation yielded the best interpretability (See Table 2). Strong relationship between the variable and the factor, it’s considered ideal loadings indicating that the factor explains a large portion (49%) of the item’s variance, moderate loadings between 0.3 and 0.7 indicating a substantial relationship and minimal loadings < 0.3 considered practically significant [49]. The structure from the exploratory factor analysis with two factors, included for the first one items B1: I have been short of breath, L4: Breathing is easy for me, L3: I feel tightness in my chest, and L1: My thinking is clear; which, according to clinical interpretability, was called respiratory symptoms and cognitive status; while the second factor included C6: I have a good appetite, L2: I have been coughing, and C2: I am losing weight, which was called cough and general symptoms (Table 2). This proposed structure explains 56% of the variance in the LCS scale.

**Table 2:** Factor loadings

Loadings		Factor 1	Factor 2	Uniqueness
B1	0.693	0.758		0.390
L4	0.571	0.715		0.484
L3	0.595	0.449	0.346	0.678
L1	0.335	0.301		0.888
C6	0.536	0.276	0.524	0.650
L2	0.387	0.231	0.313	0.848
C2	0.546		0.795	0.336

Note. Applied rotation method is quartimax. B1 I have been short of breath, C2 I am losing weight, L1 My thinking is clear, L2 I have been coughing, C6 I have a good appetite, L3 I feel tightness in my chest, L4 Breathing is easy for me

### Confirmatory factor analysis

**Fig. 1:**
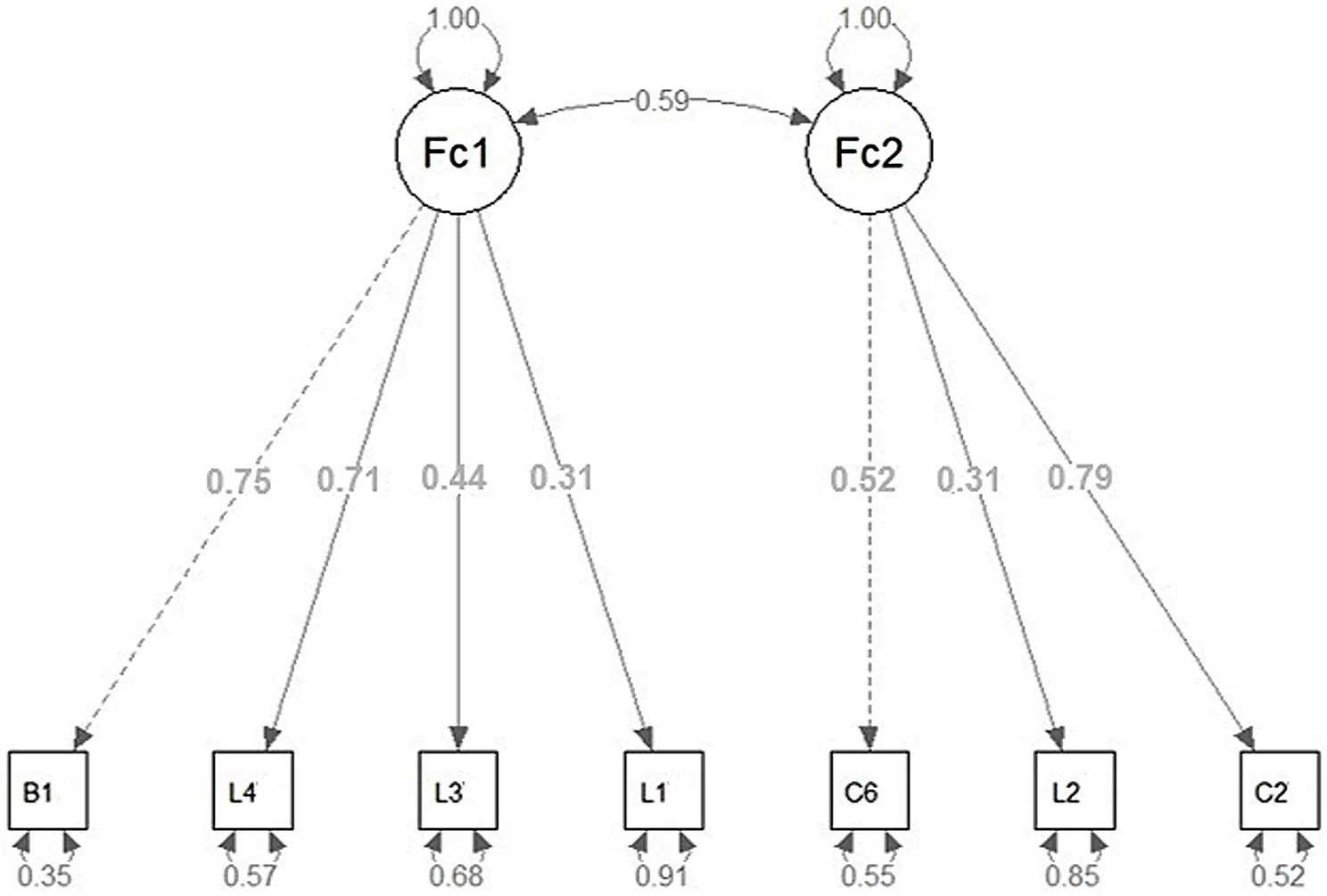
Factorial structure. Note: model having two factors

The previous factorial structure was evaluated with a confirmatory analysis and the following adjustment and error indices were found: χ² :48,5, df: 13, ( *p* <,001), RMSEA: 0,073 (90% CI % 0,039–0,108, *p* = 0,119), SRMR: 0,055, CFI: 0,981, GFI: 0,997, NNFI: 0,970, IFI: 0,975, TLI: 0,970 and MFI: 0,970. (Additional fit measures see supplemental Table 1S). The fit indices reported values that indicate a good fit of the model (Fig. 1), except for the RMSEA, it was acceptable; however, it has been reported that in small samples, the SRMR is more efficient than the RMSEA in rejecting models that do not fit closely. In this study, the SRMR value was adjusted to the suggested values [50].

### Reliability

**Fig. 2:**
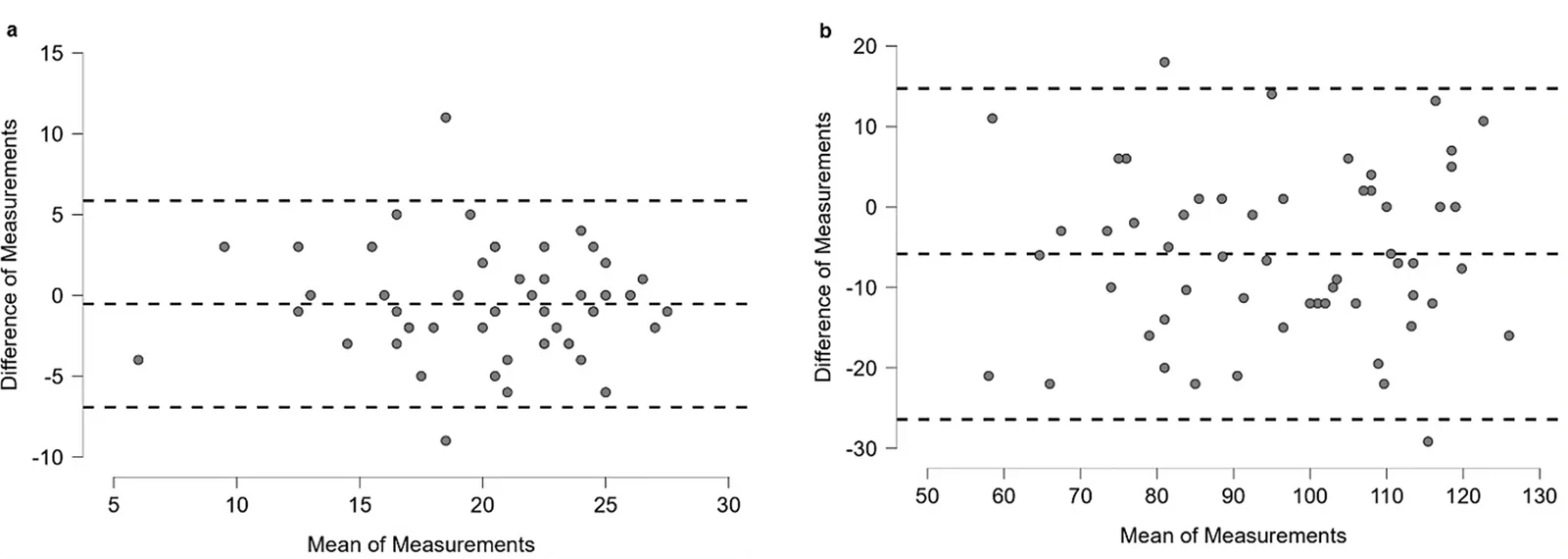
**a** LCS subscale item correlation. Note: homogeneity of variance for subscale LCS items. **b** FACT-L item correlation. Note: homogeneity of variance for FACT-L items

Test–retest reliability was assessed in a subsample of 56 participants, with an interval between 4 days and 7 days (mean 5.5 days, SD = 1.08). Lin’s concordance correlation coefficient (CCC) was used to evaluate agreement between the two administrations. The CCC value was 0.77 (95% CI 0,63 − 0,86) for the Lung Cancer Subscale (LCS), for FACT-L TOI was 0,83 (95% CI 0,73 − 0,9), and for FACT-L was 0.80 (95% CI 0,69 − 0,88) (see Table 3). These results indicate that the scale captures in its scores the temporal stability of the construct, suggesting that the FACT-L produces consistent scores when administered over time. Bland–Altman plots (Fig. 2a and b) showed no evident patterns of dispersion, further supporting the reliability of the instrument.

**Table 3:** Test-retest reliability, Means (SD), Lin's concordance-correlation coefficient and bias-correction factor

Scale	# Items	Score Range	1st Moment Mean (SD)	2nd Moment Mean (SD)	ρ c^a^	95% CI^b^	Bias-correction factor
LCS	7	0-28	19,2 (5,0)	20,8 (4,89)	0,77	0,63-0,86	0.99
FACT-L TOI	21	0-84	54,0 (14,37)	59,5 (14,14)	0,83	0,73-0,9	0.98
FACT-L	34	0-136	91,9 (19,6)	99,1 (18,49)	0,8	0,69-0,88	0.95

^a^ Lin’s concordance correlation coefficient (ρ c)

^b^ 95% confidence interval

### Internal consistency

The coefficients reported for the LCS scale were Cronbach’s alpha (α): 0.724, McDonald’s omega (ω): 0.728, Guttman’s lambda-2 (λ²): 0.73, for FACT-L TOI: 0.9, 0.91, and 0.91, for the FACT-L scale: 0,91, 0,92, and 0,92 respectively (see Table 4). Furthermore, the α and ω coefficients remained stable when recalculated with item-withdrawal procedures, suggesting that each domain’s items were reliably measuring the intended construct (see supplemental table 2S).

**Table 4:** Internal consistency assessment

Scale	# Items	Score Range	Media (SD)	Median (IQR)	α Cronbach	ω McDonald	Guttman’s λ2
LCS	7	0–28	19,2 (5,06)	20(7)	0,72	0,72	0,73
FACT-L TOI	21	0–84	54,0 (14,76)	56(23)	0,9	0,91	0,91
FACT-L	34	0-136	91,9 (19,6)	94(29)	0,91	0,92	0,92

### Convergent validity

**Fig. 3:**
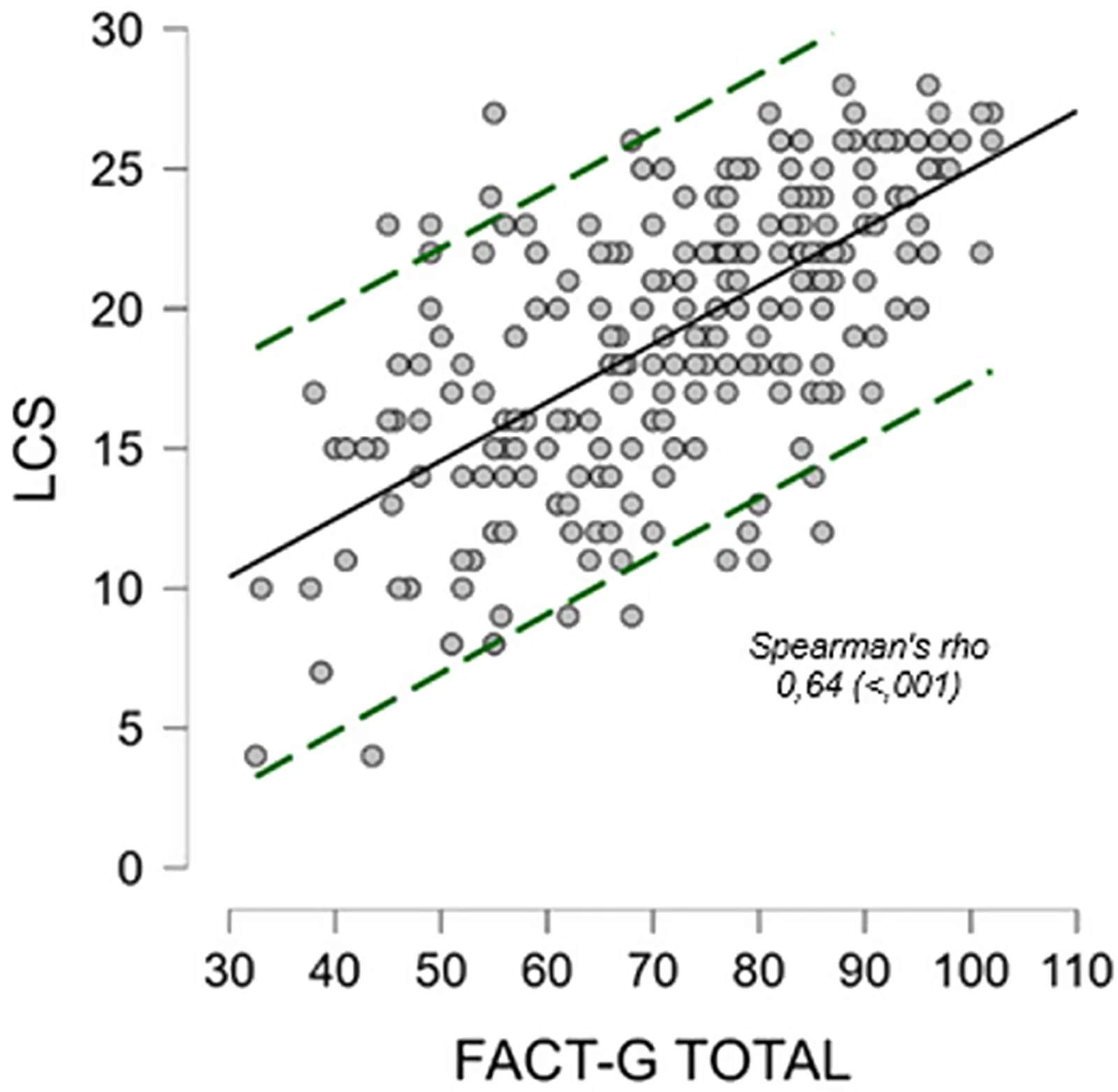
Convergent validity. Note: The correlations between LCS sub scale with FACT-G scores

This component was assessed in a separate sample of 46 patients by examining the correlation between two scales that measure the same construct, but one is a general measure (FACT-G) and the other is specific (LCS), a positive correlation is theoretically expected. Spearman’s rho correlation coefficient was 0.64 (95% CI: 0.56–0.71, *p* < 0.001), (Fig. 3).

### Divergent and discriminant validity

In divergent validity, a negative correlation is expected between ECOG and LCS, i.e., higher the Quality of Life, the lower the functionality; the result was a consistent *rho* − 0.432 (*p* < 0.001). On the other hand, in discriminant validity, it is expected that two theoretically independent constructs, such as Quality of Life and comorbidity (LCS and CCI), are not correlated; the result was a consistent rho of 0.141 (*p* = 0.027).

## Discussion

Regarding the psychometric validation of the scale, the LCS scores obtained in this study were lower than those reported in the original validation by Cella et al. [38]. However, when compared with other international studies, the score was above average. On the other hand, the overall FACT-L scores were generally consistent with published data so far, except for the Physical Well-Being (PWB) and Functional Well-Being (FWB) domains, which were slightly lower [33].

When domain scores were stratified by clinical variables, differences were observed according to ECOG performance status, Charlson Comorbidity Index (CCI), and lung cancer stage, findings that align with those reported by Aydoğan et al. [34]. This is particularly relevant in the context of lung cancer, where most patients are diagnosed at advanced stages and experience a high symptom burden, including dyspnea, cough, pain, chest tightness, and reduced functional capacity all of which negatively impact HRQoL.

In our analysis, we used different methods to determine the number of factors to extract. We discarded a 3-factor solution because it doesn’t allow for all domains to have more than 2 items. The criterion we used to choose between 1 and 2 factors was interpretability. The resulting factor structure for the remaining seven items of the Lung Cancer Subscale (LCS) demonstrated a satisfactory internal consistency, suggesting the presence of more than one underlying dimension. Other reports on factor structure are something similar, and in our case, the item L1 (My thinking is clear) showed some difficulties in the factor analysis; but instead of Saitoh et al. [32], who considered a unique item, our proposed structure with two domains showed appropriate fit and error indices in the confirmatory analyses.

On the other hand, in the study by Aydoğan et al. [34] the confirmatory factor analysis results showed that the fit to the original scale structure was within acceptable limits: the CFI was 0.917 and the RMSEA value was 0.091, but they did not report a certain structure. No additional factor analysis has been reported by validation studies published so far.

Regarding reliability, our test–retest interval averaged 5.5 days, which falls between the intervals reported in previous studies 2 weeks in Yoo et al. [30] and Franceschini et al. [33] and 1–2 days in Wan et al. [31]. Despite the slightly longer interval, the statistical and graphical methods show a good correlation between the pre- and post-test measurements, which supports the idea that if the construct is stable, the scale scores accurately reflect that stability.

Internal consistency was assessed using Cronbach’s α, McDonald’s ω, and Guttman’s λ², the results indicate that all items in the instrument are correlated and reliably measure the construct. These results exceeded those reported in the original validation by Cella et al. [38] and in subsequent studies by Saitoh [32], Wan [31] and Yoo [30], reinforcing the reliability of the Colombian version of the instrument.

Additionally, the evaluation of convergent validity demonstrated that responses across the various components of the instrument were adequately correlated, indicating a satisfactory degree of item homogeneity and internal coherence. A strong positive correlation was observed between the FACT-G and LCS scores, supporting the convergent validity of the instrument. Although the Social/Family Well-Being (SWB) and Emotional Well-Being (EWB) domains showed weaker correlations with the LCS, the overall inter-item relationships suggest that the instrument components are measuring related aspects of the same underlying construct. Divergent validity was supported by the presence of negative correlations between the LCS and ECOG performance status. Discriminant validity was also supported by the lack of correlation between The Charlson Comorbidity Index (CCI) and LCS. These findings are consistent with previous research by Aydoğan et al. [34] further confirming the scale’s ability to distinguish between conceptually distinct constructs. Although it could be considered that these scales are not sufficient to consider the concept of divergent validity.

In summary, FACT-L demonstrated a plausible factor structure, with good fit indices and low error rates, adequate levels of reliability based on its stability over time and internal consistency values, appropriate convergent, divergent, and discriminant validity. These findings support the FACT-L as a valid and reliable tool for assessing HRQoL-in patients with lung cancer in this setting. Furthermore, the number of published studies reporting validation of this scale has been in English, Chinese, Japanese, Brazilian, Korean, and Turkish languages, and the Colombian version of FACT-L becomes the first validation study in the Spanish-speaking population. Its application in clinical practice and research may facilitate future investigations into the predictive value of HRQoL scores, particularly their association with key clinical outcomes such as mortality, overall survival, and progression-free survival.

This study acknowledges limitations such as cross-sectional design and the inherent constraints of scale validation research. Using alternative approaches, such as item response theory, could provide additional data on item fit (considering difficulties with individual items), the appropriateness of the measurement scale, and the hierarchy of the items. Notably, sensitivity to change could not be evaluated due to the clinical status of the participants, many of whom were in advanced stages of disease with limited treatment options and short survival times. The regional sampling bias was because mostly Andean patients were in the sample, and the relatively small test-retest subsample. 

## Supplementary Information

Below is the link to the electronic supplementary material.

Supplementary Material 1

## Data Availability

The data were registered in the RedCap platform, with dual-entry verification by two team members to ensure data integrity. An independent monitoring team oversaw the transcription process and quality control of the dataset. https://redcap.cancer.gov.co/redcap_v7.1.2/ProjectSetup/index.php?pid=334.
