# Supplementary material for: Psychometric properties of the Functional Assessment of Cancer Therapy–Lung (FACT-L) scale to measure health-related quality of life
Source: J Patient Rep Outcomes. 2026 Jul 31;10:197. doi: 10.1186/s41687-026-01164-4 (PMC13724625; doi:10.1186/s41687-026-01164-4)
Supplement: Supplementary file 1 [file 41687_2026_1164_MOESM1_ESM.docx]

**Supplementary material**

**Table 1S Additional fit measures**

| **Index** |  |
| --- | --- |
| Comparative Fit Index (CFI) | 0.981 |
| Tucker-Lewis Index (TLI) | 0.970 |
| Bentler-Bonett Non-normed Fit Index (NNFI) | 0.970 |
| Bentler-Bonett Normed Fit Index (NFI) | 0.955 |
| Parsimony Normed Fit Index (PNFI) | 0.591 |
| Bollen's Relative Fit Index (RFI) | 0.927 |
| Bollen's Incremental Fit Index (IFI) | 0.975 |
| Relative Noncentrality Index (RNI) | 0.975 |
| **Metric** |  |
| Root mean square error of approximation (RMSEA) | 0.073 |
| RMSEA 90% CI lower bound | 0.039 |
| RMSEA 90% CI upper bound | 0.108 |
| RMSEA p-value | 0.119 |
| Standardized root mean square residual (SRMR) | 0.055 |
| Hoelter's critical N (α = .05) | 198.660 |
| Hoelter's critical N (α = .01) | 245.739 |
| Goodness of fit index (GFI) | 0.997 |
| McDonald fit index (MFI) | 0.970 |
| Akaike (AIC) | 5130.526 |
| Bayesian (BIC) | 5183.227 |

*Note:* Estimator Unweighted Least Squares (ULS), Optimization method Nonlinear Minimization subject to box constraints (NLMINB	)

|  | | | | | | | | | | | | | | | | | | | | |
| --- | --- | --- | --- | --- | --- | --- | --- | --- | --- | --- | --- | --- | --- | --- | --- | --- | --- | --- | --- | --- |
| **Table 2S Frequentist Individual Item Reliability Statistics – LCS** | | | | | | | | | | | | | | | | | | | | |
|  | | **Coefficient ω (if item dropped)** | | | | | | | **Coefficient α (if item dropped)** | | | | | | **Guttman's λ2 (if item dropped)** | | | | | |
| Item | | Estimate | | | Lower 95% CI | | Upper 95% CI | | Estimate | | Lower 95% CI | | Upper 95% CI | | Estimate | | Lower 95% CI | | Upper 95% CI | |
| **B1** |  | | 0.684 |  | 0.623 |  | 0.745 |  | 0.666 |  | 0.603 |  | 0.728 |  | 0.681 |  | 0.620 |  | 0.741 |  |
| **C2** |  | | 0.692 |  | 0.634 |  | 0.751 |  | 0.681 |  | 0.619 |  | 0.743 |  | 0.695 |  | 0.634 |  | 0.756 |  |
| **C6** |  | | 0.687 |  | 0.627 |  | 0.746 |  | 0.684 |  | 0.626 |  | 0.743 |  | 0.698 |  | 0.641 |  | 0.754 |  |
| **L1** |  | | 0.720 |  | 0.666 |  | 0.773 |  | 0.723 |  | 0.665 |  | 0.781 |  | 0.732 |  | 0.677 |  | 0.787 |  |
| **L2** |  | | 0.719 |  | 0.665 |  | 0.773 |  | 0.717 |  | 0.664 |  | 0.770 |  | 0.732 |  | 0.681 |  | 0.782 |  |
| **L3** |  | | 0.673 |  | 0.611 |  | 0.736 |  | 0.676 |  | 0.617 |  | 0.736 |  | 0.692 |  | 0.636 |  | 0.749 |  |
| **L4** |  | | 0.706 |  | 0.649 |  | 0.763 |  | 0.691 |  | 0.631 |  | 0.750 |  | 0.705 |  | 0.646 |  | 0.764 |  |
|  | | | | | | | | | | | | | | | | | | | | |

**Table 3Sa Mean and Std. deviation for scores for LCS, FACT-TOI and FACT-L according to ECOG, Charlson Comorbidity Index (CCI) and Stage scores**

|  |  | **LCS** | **FACT-TOI** | **FACT-L** | |
| --- | --- | --- | --- | --- | --- |
| ECOG  Performance Status Scale | **0** | 21,13 (4,37) | 61.54 (12,2) | 100.97 (16,4) | |
|  | **1** | 19,18 (4,39) | 51.72 (11,4) | 89.69 (16,3) | |
|  | **2** | 16,15 (4,49) | 43.05 (12,3) | 78.52 (16,1) | |
|  | **3** | 11,8 (4,89) | 30.40 (9,74) | 60.20 (14,5) | |
|  | **4** | 13.0 (7,93) | 31.00 (8,88) | 61.50 (14,2) | |
|  |  |  |  |  | |
| Charlson Comorbility  Index CCI | **Moderate ^a^** | 19.8 (5.56) | 58.4 (15.1) | 98.1 (19.9) | |
|  | **Severe** | 19.0 (4.87) | 52.5 (14.3) | 89.7 (19.0) | |
|  |  |  |  |  | |
| Lung Cancer Stage | **I** | 22.1 (4.23) | 65.5 (11.9) | 107.4 (16.2) | |
|  | **II** | 21.1 (5.64) | 60.6 (12.3) | 98.7 (16.4) | |
|  | **III** | 19.0 (4.64) | 53.9 (14.4) | 91.7 (18.9) | |
|  | **IV** | 18.6 (5.04) | 51.3 (14.3) | 88.1 (19.0) | |

**^a^** (CCI scores of 3–4)

^b^ (CCI scores ≥5)

**Figure 1S Non-Graphical solutions to Scree plot**

**Eigenvalues: factors >1 are suggested to retain**
